# Hearing Preservation and Complications of the Middle Cranial Fossa Approach for Otolaryngological Diseases: Twelve-Year Single-Center Experience

**DOI:** 10.3390/jcm14217874

**Published:** 2025-11-06

**Authors:** Toshihito Sahara, Takeshi Fujita, Yujiro Hoshi, Hajime Koyama, Anjin Mori, Yasuhiro Osaki, Akinori Kashio, Yasuhiro Sanada, Katsumi Doi

**Affiliations:** 1 Department of Otorhinolaryngology and Head and Neck Surgery, Faculty of Medicine, The University of Tokyo, Tokyo 113-8655, Japan; sahatarian@yahoo.co.jp (T.S.); harufsf@gmail.com (H.K.); a.h.cis.d.e.fis.gis.a@gmail.com (A.M.); kashioa@gmail.com (A.K.); 2 Department of Otolaryngology-Head and Neck Surgery, Kobe University Graduate School of Medicine, Kobe 650-0017, Japan; 3 Department of Otorhinolaryngology, Mitsui Memorial Hospital, Tokyo 101-8643, Japan; xin.hoshi@gmail.com; 4 Department of Otorhinolaryngology, Ikeda City Hospital, Ikeda 563-8510, Japan; aac61130@pop02.odn.ne.jp; 5 Department of Neurosurgery, Kindai University Faculty of Medicine, Osaka-Sayama 589-8511, Japan; sanada.yasuhiro@gmail.com; 6 Ear Center, Iseikai International General Hospital, Osaka 530-0052, Japan

**Keywords:** middle cranial fossa approach, vestibular schwannoma, petrous bone cholesteatoma, MCF

## Abstract

**Objectives:** The middle cranial fossa (MCF) approach is valued for preserving hearing while accessing the internal auditory canal (IAC), petrous apex, inner ear, and related structures. This study evaluated its clinical outcomes across otolaryngological diseases, focusing on postoperative complications, hearing preservation, and the effect of IAC manipulation on auditory function. **Methods:** We retrospectively analyzed 35 patients who underwent MCF otologic surgery at a single center over twelve years. We calculated the proportion of MCF cases among all otologic surgeries and assessed postoperative complications and hearing changes (bone conduction thresholds). Outcomes were compared between patients with and without IAC manipulation. **Results:** MCF procedures comprised 1.4% of all otologic surgeries. Petrous bone cholesteatoma was the leading indication (15 cases). Intracranial complications occurred in 4 patients (11.4%): seizures, epidural abscess, and cerebral infarction. Facial nerve paralysis occurred in 3 (10.7%) patients without any cerebrospinal fluid leaks. In patients without IAC manipulation, hearing functions were preserved (22.3 ± 7.8 dB HL pre- vs. 25.7 ± 9.5 dB HL postoperatively), whereas those with IAC manipulation showed significantly greater deterioration. **Conclusions:** The middle cranial fossa approach, though technically demanding and infrequently used, offers a safe and effective option across various otolaryngological diseases. This approach achieved favorable hearing preservation with a low complication rate, particularly when intradural manipulation of the IAC was not required.

## 1. Introduction

The middle cranial fossa (MCF) approach, first introduced by William House in 1961 as a surgical technique for vestibular schwannoma [1], involves opening the internal auditory canal (IAC) from the middle cranial fossa while preserving the cochlea and semicircular canals [2]. There are now three microsurgical approaches for vestibular schwannomas, namely the MCF approach, the translabyrinthine approach, and the retrosigmoid (RS) approach, each with its own set of benefits and limitations [3,4,5]. The MCF approach affords superior hearing preservation compared with the translabyrinthine approach. Furthermore, unlike the retrosigmoid route, MCF approach provides direct superior access to the entire length of the IAC.

Relatively small vestibular schwannomas, which were once regarded as well-suited for the MCF approach, are increasingly being managed with options such as Gamma Knife radiosurgery or a wait-and-scan strategy [3], resulting in a declining trend in reports on the use of the MCF approach for vestibular schwannoma. In recent years, endoscope-assisted modifications of the traditional MCF and RS approaches have been increasingly introduced in vestibular schwannoma surgery to improve visualization of the internal auditory canal while minimizing temporal lobe retraction, offering safer and less invasive alternatives [6,7,8]. These developments highlight the growing trend toward minimally invasive, endoscope-assisted skull base surgery and provide valuable context for evaluating the classical microscopic MCF approach presented in this study.

The MCF approach is one of the most versatile and important approaches in otologic surgery. It is not limited to the management of vestibular schwannoma but is also highly useful for a wide range of otolaryngological disorders, including petrous bone cholesteatoma [9,10,11], superior semicircular canal dehiscence [12,13,14,15,16], traumatic facial nerve paralysis [17,18,19], and severe Ménière’s disease for vestibular nerve section [20,21,22]. The middle fossa route affords direct extradural access to the IAC and adjacent targets—including petrous apex, superior semicircular canal, and the superior tympanic cavity around the geniculate ganglion and labyrinthine facial nerve. This anatomical reach underlies its use across diverse otologic diseases where preservation of hearing and facial nerve function is desired.

Although the MCF approach has been widely reported in the context of individual diseases, such as vestibular schwannoma and petrous bone cholesteatoma, relatively few reports comprehensively analyze surgical outcomes of MCF approaches across multiple diseases in otolaryngology. Reports focusing on a single disease provide valuable insights, but they may not fully capture the broader utility of the MCF approach as a surgical technique in otolaryngology. Such studies also do not clarify how frequently the MCF approach is performed, how risky it is, or how important it is within the field. Therefore, this study comprehensively evaluates the frequency and outcomes of the MCF approach across various otolaryngological diseases, with a particular focus on postoperative complications and hearing preservation, aiming to identify the diseases and conditions in which the MCF technique is most beneficial.

The MCF approach also requires a large incision and craniotomy, excessive temporal lobe retraction, and a longer recovery time than the transmastoid and transcanal approaches commonly used in otologic surgery. It can lead to various intracranial complications, such as cerebrospinal fluid leak, meningitis, and temporal lobe injury, so the support of neurosurgeons is essential [5,23,24]. Additionally, the MCF approach provides access to various regions of the temporal bone, including the IAC, petrous apex, inner ear, and superior tympanic cavity. Precise manipulation is significant when handling within the IAC, where the facial nerve, cochlear nerve, and vestibular nerves are closely located, because of the increased risk of serious neurological complications.

## 2. Materials and Methods

### 2.1. Patients

From April 2010 to March 2022, we retrospectively reviewed patients who underwent surgery using the middle cranial fossa (MCF) approach at the Department of Otolaryngology, Kindai University Hospital. This study was approved by the regional ethical standards committee (27–110). This study examined the number of MCF surgeries, their proportion among total otologic surgeries under general anesthesia, and patient characteristics, including age, sex, and underlying diseases. For petrous bone cholesteatoma cases, the classification and extent of intraoperative involvement [9,10,25] were investigated. Surgical decisions were made according to clinical guidelines and through consultation between otolaryngology and neurosurgery.

### 2.2. Inclusion and Exclusion Criteria

This retrospective study included all patients who underwent MCF surgery for various otolaryngological diseases during a continuous 12-year period, corresponding to the tenure of two senior surgeons (one otologic and one neurosurgical) at our institution. During this period, all MCF procedures were performed by this dedicated surgical team, and no additional inclusion or exclusion criteria were applied. Our institution is a university hospital capable of managing all otologic procedures, and there were no cases referred to other hospitals due to surgical complexity or complications.

### 2.3. Surgical Procedures and Techniques

Patients were positioned supine with the head rotated contralaterally and secured in a standard three-pin neurosurgical head holder. Intraoperative facial nerve monitoring was performed in all cases using a NIM system (Medtronic, Minneapolis, MN, USA). Bipolar stimulation (0.5–1.0 mA) was used to identify and monitor the facial nerve, and electromyographic (EMG) responses were recorded from the orbicularis oculi and orbicularis oris muscles via needle electrodes. For all MCF procedures, a lumbar drain was placed preoperatively with the assistance of neurosurgeons to reduce intracranial pressure and to facilitate temporal lobe retraction. Although not universally required for this approach, lumbar drainage was our institution’s standard protocol. The dura mater was carefully detached and elevated from the skull base toward the target area within the MCF. These steps were consistently performed by the neurosurgeon using the same procedure for each case. Subsequently, the main surgical procedures, such as tumor excision or cholesteatoma removal, were performed by the otolaryngologist. At the end of the surgery, the neurosurgeon closed the cranial window after confirming the absence of Cerebrospinal fluid (CSF) leak or persistent active bleeding. Postoperatively, all patients were hospitalized in the intensive care unit (ICU) for at least one day, and fosphenytoin was often administered for seizure prevention [26,27]. Bed rest was maintained during this period. Postoperative head CT scans were performed in all patients on the first day after surgery in the ICU to rule out intracranial complications. After removal of the lumbar drain on the second postoperative day, no specific restrictions on head or body position were applied unless symptoms occurred. This is shown in Supplementary Data S1.

### 2.4. Surgical Team Consistency

All MCF surgeries were performed under the supervision of the same senior otologic surgeon (K.D.), who led in all cases. Each operation involved one or two neurosurgeons, with the same lead neurosurgeon (Y.S.) performing all craniotomies throughout the study period. Although assistant surgeons and residents rotated—with more than ten residents participating over the 12-year period—the core team, including anesthesiologists and nurses, remained largely consistent. The lead otologic surgeon had extensive prior experience with the MCF approach, having performed numerous cases over nearly two decades at a university-affiliated tertiary referral center before the study period.

### 2.5. Assessment of Postoperative Complications

The type and frequency of postoperative complications were examined for each disease. Postoperative complications were defined as those newly occurring within seven days after surgery, including cases that subsequently improved. The CSF leaks were evaluated by postoperative rhinorrhea. All patients underwent a head CT scan on the day after surgery to rule out intracranial complications such as hemorrhage. Complications present before surgery were excluded. Postoperative care for all cases involved collaborative management by otolaryngologists and neurosurgeons, and both departments conducted follow-up for complications.

### 2.6. Audiometric Assessment

The preservation of hearing, which is one of the significant advantages of the MCF approach, was assessed using the bone conduction threshold in pure-tone audiometry. Because this study included multiple diseases and considered the varied conditions of the middle ear and the presence or absence of ossicular reconstruction, the bone conduction threshold was suitable for evaluating hearing preservation. The difference in average bone conduction thresholds (250, 500, 1000, 2000, 4000 Hz) between preoperative and postoperative periods was used to assess hearing. For the analysis of hearing preservation, patients were divided into two groups according to whether intradural manipulation of the IAC was performed. Details of this classification are provided in Supplementary Data S2.

### 2.7. Statistical Analysis

Data management was performed using Microsoft Excel (version 16.75.2 for iOS; Microsoft, Redmond, WA, USA), and statistical analyses were conducted with GraphPad Prism 9 (GraphPad Software, Boston, MA, USA). Descriptive data are presented as mean ± standard deviation (SD). The distribution of continuous variables was assessed using the Shapiro–Wilk test to confirm the appropriateness of parametric testing. As the data were approximately normally distributed but exhibited unequal variances between groups, Welch’s *t*-test (*t*-test with unequal variance correction) was employed for group comparisons. All statistical analyses were two-tailed, and a *p*-value < 0.05 was considered statistically significant.

## 3. Results

### 3.1. Patient Demographics and Disease Distribution

**Figure 1:**
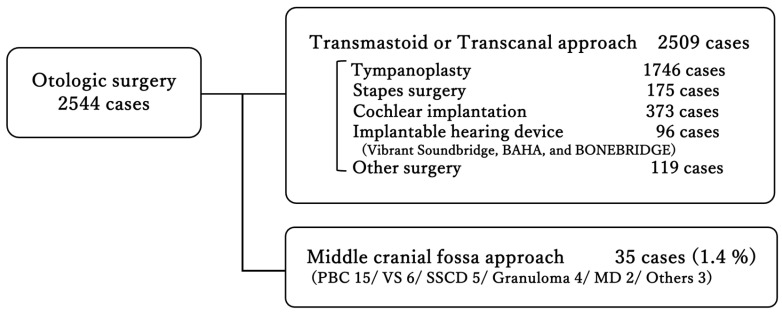
Distribution of otologic surgeries performed at a single center over twelve years. The majority were conducted via transmastoid or transcanal approaches, whereas only 1.4% required a middle cranial fossa approach. PBC, Petrous Bone Cholesteatoma; VS, Vestibular Schwannoma; SSCD, Superior Semicircular Canal Dehiscence; Granuloma, Cholesterol Granuloma; MD, Ménière’s disease.

Over the past twelve years, 35 of 2544 otologic surgeries (1.4%) were performed using the MCF approach (Figure 1). Among these, Petrous Bone Cholesteatoma was the most common with 15 cases, followed by Vestibular Schwannoma and Superior Semicircular Canal Dehiscence. Inflammatory diseases, Petrous Bone Cholesteatoma, and granuloma together accounted for 19 cases, representing more than half of the total (Table 1). The sex distribution was 18 males and 17 females, with an average age of 47.4 ± 17.3 years (range: 11–73 years). The two youngest cases, in their teens, both presented with Petrous Bone Cholesteatoma. In the classification of petrous bone cholesteatoma, the supralabyrinthine type was the most common in seven cases, followed by the apical type in four cases (Supplementary Data S3). Regarding the extent of intraoperative involvement of petrous bone cholesteatoma, the dura mater was involved in the majority of cases, followed by the semicircular canal and internal acoustic meatus (Supplementary Data S4).

### 3.2. Postoperative Complications

Postoperative intracranial complications included cerebral abscess, seizures, infarction, and facial palsy, and no cases of CSF leak were observed (Table 2). Intracranial complications were observed in four cases: one petrous bone cholesteatoma and three cholesterol granulomas. Postoperatively, two seizure episodes were observed, consisting of one absence-like event and one convulsive event. One case of epidural abscess required revision surgery for infection control. Headaches occurred in five cases (22.7%), all of which developed in the immediate postoperative period. All cases were associated with petrous bone cholesteatoma. Concerning facial palsy and headache, cases in which these symptoms were present preoperatively were excluded. Postoperative complications of facial nerve paralysis occurred in three cases (10.7%), including two cases of vestibular schwannoma and a case of giant cell tumor. In all three cases, the degree of facial nerve palsy was House-Brackmann grade III, and it developed immediately after surgery. Postoperative head CT scans performed in all patients on the first day after surgery in the ICU showed no evidence of abnormalities such as brain contusion, intracranial hemorrhage, or cerebral edema.

### 3.3. Hearing Preservation

**Figure 2:**
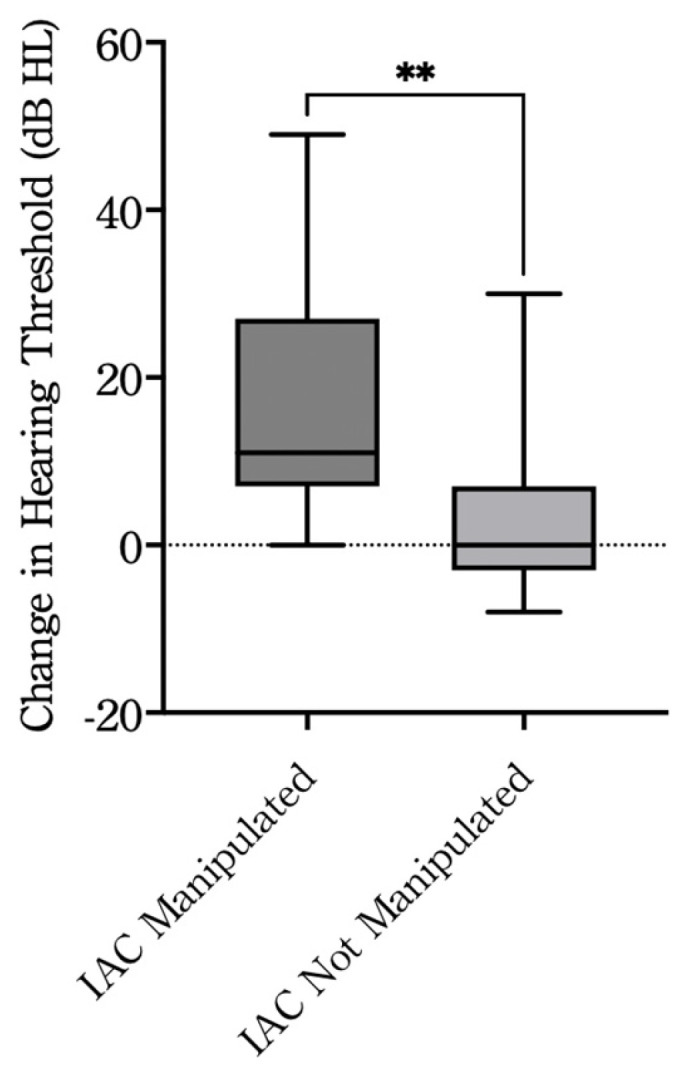
Change in hearing thresholds between pre- and postoperative evaluations. IAC Manipulated: cases with surgical manipulation of the internal auditory canal (*n* = 15); IAC Not Manipulated: cases without manipulation (*n* = 19). The dashed horizontal line indicates no change in hearing threshold (Δ = 0). ** *p* < 0.01, Welch’s *t*-test. dB, decibel; HL, hearing level; IAC, internal auditory canal.

The average bone conduction (BC) thresholds were 26.0 ± 9.1 dB HL before surgery and 35.2 ± 10.0 dB HL after surgery in all cases. Of the 35 patients, postoperative audiometric evaluation was not performed in one case, and hearing outcomes were analyzed in the remaining 34 patients. When cases were divided based on surgical manipulation within the IAC, the BC thresholds in the non-manipulation group (*n* = 19) were 22.3 ± 7.8 dB HL before surgery and 25.7 ± 9.5 dB HL after surgery. In contrast, in the manipulation group (*n* = 15), the BC thresholds were 30.7 ± 11.8 dB HL before surgery and 47.2 ± 13.2 dB HL after surgery (Table 3). The change in BC thresholds from before to after surgery was 3.4 ± 9.0 dB HL in the non-manipulation group and 16.5 ± 12.6 dB HL in the manipulation group. A statistically significant difference was observed between the two groups (*p* < 0.01) (Figure 2), indicating that BC hearing was significantly better preserved in the non-manipulation group.

## 4. Discussion

The middle cranial fossa (MCF) approach, initially reported for vestibular schwannoma surgery, has proven to be a valuable technique in otolaryngology and has been applied not only to vestibular schwannomas but also to other diseases such as petrous bone cholesteatoma, Superior semicircular canal dehiscence, and facial nerve paralysis. This approach can access the petrous apex and internal auditory canal (IAC) while preserving inner ear functions, making it a crucial procedure. While the MCF approach is relatively uncommon in otologic surgery, our study, accounting for 1.4% of cases, underscores its significance. In addition, no one has previously reported on the percentage of MCF approaches to all otologic surgeries, including implantable hearing devices. Among the disease entities, petrous bone cholesteatoma was the most frequent indication, accounting for 42.9% of cases. The classification and extent of intraoperative involvement of petrous bone cholesteatoma observed in this study were consistent with previous reports, with the supralabyrinthine type being the most common [9,11,25]. Dura mater involvement was also frequently observed, which may be attributed to the anatomical characteristics of supralabyrinthine type. These findings suggest that MCF is an appropriate approach for accessing and treating supralabyrinthine type, as well as for minimizing unnecessary damage to surrounding structures.

In addition to the MCF approach, retrosigmoid (RS) and translabyrinthine approaches to the IAC are also available. Still, each technique has a different pathway and therefore different advantages and limitations. The RS approach requires a craniotomy and cerebellar retraction. While it provides a good view of the cerebellopontine angle and allows for the removal of large vestibular schwannomas, it is less suitable for small tumors confined to the IAC. The translabyrinthine approach follows the same surgical route commonly used by otolaryngologists in ear surgery. However, since it necessitates drilling through the inner ear to access the IAC, hearing preservation is generally not feasible. The MCF approach involves superior retraction of the temporal lobe, allowing access to nearly the full length of the IAC while preserving hearing among patients with small tumors. Although this approach carries a risk of temporal lobe injury and a higher likelihood of facial nerve palsy than other approaches, it may offer potential benefits in hearing preservation, particularly in selected cases. Our findings support its clinical relevance, especially in cases where preservation of hearing and facial nerve function are key surgical priorities. The characteristics of each surgical approach for vestibular schwannoma, including complications, surgical procedures, and access, based on previous reports [3,4,5], are summarized in Table 4.

Facial nerve paralysis is one of the most serious complications associated with the MCF approach. Compared to the RS and translabyrinthine approaches, the MCF approach has been reported to have higher rates of facial nerve paralysis [3]. The reported incidence of facial nerve palsy following MCF surgery for intracanalicular vestibular schwannomas ranges from 19% to 26%, depending on tumor size [24,28,29]. In this study, the incidence of postoperative facial paralysis was 10.7% (three cases), which is notably lower than in previous reports. When focusing on vestibular schwannoma alone, the incidence was 33% (two cases), which is higher than that reported in the literature. This discrepancy may be explained by the relatively large tumor sizes in these two patients (16.6 mm and 9.2 mm) as well as the limited sample size of only six vestibular schwannoma cases. In contrast, in other pathologies such as petrous bone cholesteatoma and superior semicircular canal dehiscence, the incidence of postoperative facial nerve palsy was almost negligible when no preoperative palsy was present. This result may be attributed to meticulous surgical techniques, including the use of intraoperative facial nerve monitoring, as well as the expertise of the surgical team. These included two cases of vestibular schwannomas and one case of a giant cell tumor. One of the two vestibular schwannomas showed improvement (House-Brackmann I), while the other remained paralyzed (House-Brackmann III). The patient with the giant cell tumor had undergone two prior tumor resection surgeries at other hospitals. Permanent facial paralysis due to surgery was observed in only two cases (7.1%).

Intracranial complications such as cerebrospinal fluid (CSF) leak, meningitis, and cerebrovascular disease are potential risks of the MCF approach. CSF leak is one of the most common complications among them, with reported rates ranging from 5.3% to 13% [5,23,30,31]. In this study, there were 15 cases of repaired dura mater, including cases of diseases other than vestibular schwannoma, but none had CSF leak as a complication. It may be attributed to our repair techniques, including adequate CSF pressure control, use of fascia, and reinforcement with bone paste and fibrin glue. Careful verification by an otolaryngologist and neurosurgeon during the procedure may also have contributed to the low complication rate. Furthermore, the lead otologic surgeon had already gained extensive experience with the MCF approach before the beginning of this study, having performed numerous such procedures over nearly two decades. Throughout the present series, the same neurosurgeon served as the primary operator for all cases. No temporal clustering of complications was observed, suggesting that variations in team composition or the learning curve did not influence surgical outcomes.

Other complications, such as epidural abscess, cerebral infarction, and seizure, were observed in a few cases and were more frequent in patients with extensive lesions or prior surgeries. One patient with petrous bone cholesteatoma who developed an epidural abscess had previously undergone surgery for an extensive cholesteatoma at another hospital, and the present procedure was a reoperation for residual disease to control the abscess infection. Another patient who developed postoperative cerebral infarction already had destruction of the semicircular canal due to the lesion, and the infarction occurred on the contralateral side of the operation, making a direct causal relationship with the intraoperative manipulation unlikely. A convulsive seizure occurred once on the day of surgery, and an absence-like seizure was observed as a transient episode. Such transient seizures are recognized complications after craniotomy, often related to cortical irritation or temporal lobe retraction. In our series, two patients developed these episodes (one convulsive and one absence-like), both occurring only once without recurrence. These events are not uncommon in neurosurgical procedures and may be minimized by perioperative seizure prophylaxis, such as the routine administration of fosphenytoin in our institution. Those four cases were all petrous apex lesions, and two of them had very extensive lesions. The risk of intracranial complications may be higher in cases that are reoperations or have more extensive lesions.

Hearing preservation is one of the most critical outcomes in the MCF approach, a technique known for its hearing-preserving potential. In vestibular schwannoma, tumor size is a key factor influencing postoperative hearing outcomes, with larger tumors associated with a higher risk of hearing loss [3]. In a systematic review, the hearing preservation rate following MCF surgery for tumors smaller than 15 mm was 56.4%, whereas it was only 17.3% for tumors 15 mm or larger [15] using AAO-HNS Committee on Hearing and Equilibrium classification [32]. The average tumor size in this study was 11.3 ± 1.3 mm [7.0–16.6 mm], and our hearing preservation rate was 50.0%, which is comparable to the rates reported in previous studies using the AAO-HNS classification.

In comparison to the natural course of these diseases, several studies have investigated hearing outcomes in vestibular schwannoma (VS) managed conservatively with a “wait-and-scan” strategy. Vestibular schwannoma is a slowly growing tumor that gradually leads to progressive hearing deterioration, although its growth rate is generally not rapid. Prasad et al. analyzed 576 VS patients and found that 56.1% of those with serviceable hearing at baseline maintained it after five years of observation [33]. Similarly, Khandalavala et al. reported that among 157 patients initially having serviceable hearing, 94%, 81%, and 78% retained it at 1, 3, and 5 years, respectively [34]. In another large cohort, Stangerup et al. found that 59% of patients preserved serviceable hearing at 4–5 years, and 69% maintained it for more than 10 years without intervention [35]. These findings indicate that even under observation, hearing gradually deteriorates over time in a substantial proportion of VS patients. In our series, among six VS patients, two had initially been observed for over two years before progressive hearing loss and tumor enlargement led to surgery. Postoperatively, hearing preservation was achieved in three of six cases (50%), which is comparable to, or slightly worse than, the long-term hearing outcomes reported in the literature for non-surgical cohorts. Similarly to VS, Ménière’s disease (MD) also exhibits progressive hearing deterioration during its natural course, and therefore, hearing outcomes following surgery are sometimes compared with those of long-term conservative follow-up. In the present series, the surgical indication for MD was primarily for intractable vertigo rather than hearing improvement. Of the two MD cases, one already had profound hearing loss before surgery, while the other maintained moderate hearing levels (51.3 dB HL preoperatively and 62.5 dB HL postoperatively; 500–4000 Hz average) following middle fossa vestibular neurectomy. Previous studies have shown that this approach can achieve favorable hearing preservation even in advanced MD, with more than half of patients maintaining their preoperative levels [36]. In contrast, longitudinal follow-up of conservatively treated MD has revealed that although most cases stabilize after approximately four years, around 20% follow a relapsing course with progressive hearing deterioration and frequent Tumarkin attacks [37]. These findings suggest that the MCF approach may provide adequate vertigo control without accelerating the natural progression of hearing loss in MD.

Importantly, in cases other than vestibular schwannoma, hearing was generally preserved. Our analysis revealed that when intradural manipulation within the IAC was not required, postoperative hearing was well maintained in patients with petrous bone cholesteatoma, superior semicircular canal dehiscence, and traumatic facial palsy. In contrast, cases involving IAC manipulation were associated with significantly worse postoperative hearing outcomes. Of the 15 patients who underwent IAC manipulation, a giant cell tumor was massive and involved a wide area within the IAC, and half of petrous bone cholesteatoma cases were reoperations, which may have caused the postoperative deterioration of hearing. These results align with previous reports, which have suggested that IAC invasion in petrous bone cholesteatoma reduces the likelihood of hearing preservation regardless of the surgical approach [38]. Our findings further emphasize the importance of surgical strategy and the potential to optimize outcomes by minimizing IAC manipulation.

Recently, endoscope-assisted microsurgical approaches have been increasingly adopted in lateral skull base surgery to enhance visualization and minimize temporal lobe retraction. Several groups have described endoscope-assisted modifications of both the MCF and RS approaches for vestibular schwannoma resection. For example, Master et al. and Moon et al. reported that endoscope-assisted MCF techniques improved visualization of the fundus of the internal auditory canal and reduced the need for extensive temporal lobe retraction [6,7]. Similarly, Bi et al. demonstrated that endoscopic assistance during the RS approach enabled better identification of residual tumor within the internal auditory canal [8]. More recently, Hosoya et al. reported favorable hearing-preservation results using an endoscope-assisted retrolabyrinthine approach under continuous intraoperative monitoring [39]. Although our current series did not utilize endoscopic assistance, the favorable hearing preservation and low complication rate observed here provide a valuable reference for comparison with these emerging minimally invasive, endoscope-assisted techniques.

In summary, our findings highlight that the middle cranial fossa approach, though technically demanding and rare in otologic surgery, can serve as a safe and reliable option across multiple diseases, achieving favorable hearing outcomes with a low complication rate, particularly when IAC manipulation is unnecessary.

This study has several limitations. First, it included heterogeneous disease entities with distinct pathophysiological mechanisms and surgical objectives, which may have influenced both the complication profile and hearing outcomes. Second, the relatively small subgroup sizes limited the statistical power for detailed comparisons among disease categories. Third, the follow-up period was relatively short, and long-term hearing outcomes could not be fully evaluated, particularly for slowly progressive pathologies such as vestibular schwannoma and Ménière’s disease. Future multicenter prospective studies with standardized audiometric protocols and extended observation periods are warranted to validate our findings. Finally, as a retrospective study, uniform speech perception data were not available across all pathologies. In particular, for petrous bone cholesteatoma and other inflammatory diseases, speech audiometry was seldom performed because postoperative hearing was generally stable. Therefore, overall hearing preservation was evaluated using bone-conduction thresholds, which provide an accurate representation of cochlear function independent of conductive components.

## 5. Conclusions

In conclusion, the middle cranial fossa approach, although technically demanding, provides a safe and effective surgical option for selected otolaryngological diseases. It achieves favorable hearing preservation with a low rate of serious complications, particularly in cases where intradural manipulation of the IAC is not required.

## Figures and Tables

**Table 1:** Demographic and clinical characteristics.

Characteristic	Value
Sex (Male/Female)	18/17
Mean Age (range)	47.4 ± 17.3 (11–73)
Surgical Side (Right/Left)	9/26
Etiology	
Cholesteatoma	15
Vestibular schwannoma	6
Superior canal dehiscence syndrome	5
Cholesterol granuloma	4
Ménière’s disease	2
Traumatic facial palsy	1
Facial schwannoma	1
Giant cell tumor	1

Values are presented as the number of cases. Mean values are shown with standard deviations (SD) where applicable.

**Table 2:** Postoperative complications following middle cranial fossa surgery.

Postoperative Complications						
	Abscess	Seizure	Cerebral Infarction	CSF Leak	Facial Palsy	Headache
	*n* = 1 (1/35)	*n* = 2 (2/35)	*n* = 1 (1/35)	*n* = 0 (0/35)	*n* = 3 (3/28)	*n* = 5 (5/22)
Petrous bone cholesteatoma	1	0	0	0	0	5
Vestibular Schwannoma	0	0	0	0	2	0
Granuloma	0	2	1	0	0	0
SSCD	0	0	0	0	0	0
Ménière’s disease	0	0	0	0	0	0
Others	0	0	0	0	1 *	0

Values are presented as the number of cases (n) or n/total. No cerebrospinal fluid (CSF) leaks were observed. SSCD, superior semicircular canal dehiscence; CSF, cerebrospinal fluid. *: Giant cell tumor.

**Table 3:** Comparison of hearing outcomes between cases with and without surgical manipulation of the internal auditory canal.

		Preoperative Values	Postoperative Values	Statistical Significance
		Mean ± SD (dB HL)	Mean ± SD (dB HL)	*p* Value
Surgical manipulation of IAC	+ (*n* = 15)	30.7 ± 11.8	47.2 ± 13.2	<0.001
	− (*n* = 19)	22.3 ± 7.8	25.7 ± 9.5	0.116
Total (*n* = 34)		26.0 ± 9.1	35.2 ± 10.0	<0.001

Values are presented as mean ± SD. Bone-conduction thresholds represent the average of six tested frequencies (0.25–8 kHz). *p*-values were calculated using Welch’s *t*-test. dB, decibel; HL, hearing level; IAC, internal auditory canal.

**Table 4:** Characteristics of Approaches for Vestibular Schwannoma.

	Characteristics of Approaches for Vestibular Schwannoma		
	Middle Cranial Fossa	Translabyrinthine	Retrosigmoid
Surgical Procedure	Craniotomy Temporal lobe retraction	Inner ear sacrifice Fat graft	Craniotomy Cerebellar retraction Intradural drilling of the IAC
Access	Full length of the IAC Limited CPA angle access	Full length of the IAC and fundus Wide posterior fossa exposure	CPA-angle exposure Limited access to the lateral IAC
Complications	Possible hearing preservation Risk of facial nerve paralysis, and intracranial complications	Inevitable hearing loss Low rate of headache	Possible hearing preservation Risk of intracranial complications

IAC, inner auditory canal; CPA, Cerebellopontine angle.

## Data Availability

Data pertaining to this study can be shared upon request to the corresponding author.

## Supplementary Materials

The following supporting information can be downloaded at https://www.mdpi.com/article/10.3390/jcm14217874/s1, Data S1: Protocol of surgery with the middle cranial fossa approach; Data S2: Classification based on surgical manipulation within the IAC; Data S3: Classification of petrous bone cholesteatoma; Data S4: Extent of intraoperative involvement of petrous bone cholesteatoma.

## Abbreviations

The following abbreviations are used in this manuscript:

BC	Bone conduction
CPA	Cerebellopontine angle
CSF	Cerebrospinal fluid
IAC	Internal auditory canal
ICU	Intensive care unit
MCF	Middle cranial fossa
PBC	Petrous bone cholesteatoma
SSCD	Superior semicircular canal dehiscence
VS	Vestibular schwannoma
