# Supplementary material for: Hearing Preservation and Complications of the Middle Cranial Fossa Approach for Otolaryngological Diseases: Twelve-Year Single-Center Experience
Source: J Clin Med. 2025 Nov 6;14(21):7874. doi: 10.3390/jcm14217874 (PMC12607951; doi:10.3390/jcm14217874)
Supplement: Supplementary file 1 [file jcm-14-07874-s001.zip › jcm-3908421-supplementary.pdf]

Supplementary Data S1. Protocol of surgery with the middle cranial fossa approach

Craniotomy

i)Neurosurgeon Part
- Placing spinal drain to reduce CSF pressure
- Dural elevation and exposure of the floor of the middle cranial fossa

⇩

Otologic Surgery in the MCF approach

ii)Otologist Part
- Dissection of the IAC
- Exposure of superior semicircular canal
- Removal of tumor /cholesteatoma /granuloma
- Repairing dura matter and MCF floor defect

⇩

MCF Window Closing

iii)Neurosurgeon Part
- Checking for CSF leak out and bleeding
- MCF window closing

⇩

ICU (for one day)

iv)Postoperative care
- CT scan
- Removing spinal drain
- Fosphenytoin

Supplementary Data S2. Classification based on surgical manipulation within the IAC

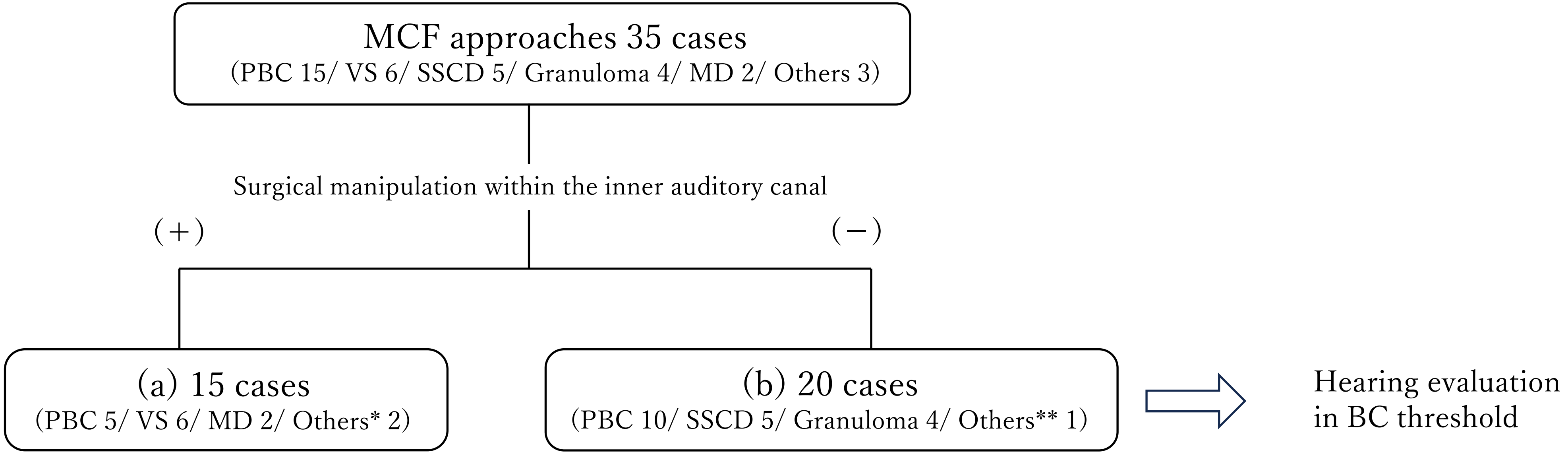

*; Giant cell tumor, Facial schwannoma
**; Traumatic facial palsy

Supplementary Data S3. Classification of petrous bone cholesteatoma

Petrous bone cholesteatoma type	Patients, *n* (%)
Supralabyrinthine	7 (47)
Infralabyrinthine	0
Infralabyrinthine-apical	1 (7)
Massive	3 (20)
Apical	4 (27)

Supplementary Data S4. Intraoperative extension of petrous bone cholesteatoma

Eroded structure	Patients, *n* (%)
Dura matter	12 (80)
Semicircular canal (s)	6 (40)
Internal acoustic meatus	5 (33)
Cochlear	4 (27)
Carotid canal	3 (20)
Intracranial compartment	0
